# Flight capacity drives circadian patterns of metabolic rate and alters resource dynamics

**DOI:** 10.1002/jez.2598

**Published:** 2022-04-19

**Authors:** Zachary R. Stahlschmidt

**Affiliations:** ^1^ Department of Biological Sciences University of the Pacific Stockton California USA

**Keywords:** cricket, food, *Gryllus*, life history, wing dimorphism

## Abstract

Animals must acquire, use, and allocate resources, and this balancing act may be influenced by the circadian clock and life‐history strategy. Field (*Gryllus*) crickets exhibit two distinct life‐history strategies during early adulthood—flight‐capable females invest in flight muscle at a cost to ovary mass, whereas flight‐incapable females instead invest solely into ovaries. In female *Gryllus lineaticeps*, I investigated the role of life‐history strategy in resource (food) acquisition and allocation, and in circadian patterns of energy use. Flight capacity increased the standard metabolic rate (SMR) due to greater late‐day SMR and flight‐capable crickets exhibited greater circadian rhythmicity in SMR. Flight‐capable crickets also ate less food and were less efficient at converting ingested food into body or ovary mass. Thus, investment into flight capacity reduced fecundity and the amount of resources available for allocation to other life‐history traits. Given the increasing uncertainty of food availability in many global regions, work in *Gryllus* may clarify the important roles of food and circadian patterns in life‐history evolution in a changing world.

## Research Highlights

In a field cricket, investment into flight capacity (1) increased the circadian rhythmicity of resource use (standard metabolic rate), (2) reduced resource acquisition (food intake), and (3) reduced the efficiency by which ingested food was converted to reproductive tissue.

## Graphical abstract

Wing‐dimorphic crickets offer unique insight into the costs and benefits of flight capacity. Here I show that flight capacity increases the circadian rhythmicity of metabolic rate, and I further show that flight‐capable crickets eat less food and are less efficient at converting ingested food into body or ovary mass. In sum, investment into flight capacity in a field cricket can represent a risky energetic strategy that reduces fecundity and the amount of resources available for allocation to other life‐history traits.

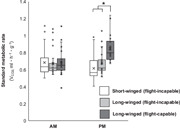

## 1 INTRODUCTION

The circadian clock in animals dictates virtually all levels of organismal biology—from gene expression and cell division to growth, behavior, and reproduction (Hori et al., 1995; Jakobsen & Strom, 2004; Panda et al., 2002; Sen & Hoffmann, 2020; Xu et al., 2011; Yeung & Naef, 2018). Many animals exhibit circadian patterns of metabolic rate (reviewed in Ellis & Gabrielsen, 2019; Mortola & Lanthier, 2004) as endogenous daily rhythms are synchronized to match the energy needs of animals (reviewed in Riede et al., 2017). Variation in metabolic rate may also be linked to life history (Bronikowski & Vleck, 2010; Crnokrak & Roff, 2002; Ksia̧zek et al., 2004; but see Clark et al., 2016; Djawdan et al., 1996, 1997). In addition, the evolution of circadian clocks may be entwined with life‐history evolution—circadian clocks and life‐history traits respond to similar selective pressures and selection on life‐history traits influences the evolution of circadian clocks (reviewed in Abhilash & Sharma, 2016). However, the interplay between life history and circadian rhythm related to metabolic rate is not fully understood.

Field crickets (*Gryllus* spp.) may offer important insight into the roles of life history and circadian clocks in standard metabolic rate (SMR), which is the energy cost of self‐maintenance and key to animal energetics (Glazier, 2005; Lighton, 2008). *Gryllus* crickets exhibit a wing dimorphism mediating two distinct life‐history strategies during early adulthood—long‐winged, flight‐capable (LW[f]) females invest in flight muscle at a cost to ovary mass, whereas short‐winged (SW) females are flight‐incapable but invest more heavily into ovaries than LW(f) females (Roff, 1984; Zera & Mole, 1994; Zera et al., 1997; Zera, 2005). The role of life‐history strategy in SMR for *Gryllus* is equivocal. Some work indicates that flight capacity is particularly costly (i.e., flight‐capable females exhibit greater SMR than SW females; Nespolo et al., 2008; Sun et al., 2020; Zera & Mole, 1994; Zera et al., 1997). Yet, other work finds no effect of flight capacity on SMR in *Gryllus* (Clark et al., 2016), or an effect only in particular contexts (e.g., flight capacity interacts with immune challenge to influence SMR; Stahlschmidt & Glass, 2020). A deeper dive into *Gryllus* physiology may reconcile these contrasting results. For example, flight capacity influences circadian physiology where flight‐capable females tend to exhibit larger daily fluctuations (i.e., greater circadian rhythmicity) in juvenile hormone levels and global gene expression patterns characterized by relative peaks late in the photophase (Zera et al., 2018; Zhao & Zera, 2004), putatively in preparation for nocturnal flight activity. As juvenile hormone increases SMR in other insects (Shigler et al., 2021), flight‐capable *Gryllus* may exhibit greater SMR late in the photophase. However, the link between variation in gene expression and SMR due to life‐history strategy is not understood in *Gryllus*. In garter snakes, fast‐aging individuals exhibit higher metabolic rate than their slow‐living conspecifics, and they exhibit higher expression of some (but not all) mitochondrial genes (Schwartz et al., 2015). Thus, further investigation is required to determine whether flight‐capacity in *Gryllus* increases circadian rhythmicity in whole‐animal resource use (i.e., SMR) similar to its effects on levels of hormones and gene expression.

In addition to circadian patterns of resource use, variation in resource acquisition and/or allocation may characterize flight‐related metabolic strategies. For example, LW(f) *Gryllus* are less efficient at converting digested food into body mass, potentially indicating a metabolic cost of flight capacity (Mole & Zera, 1993, 1994). Yet, LW crickets may offset greater energy use by exhibiting higher rates of food consumption, which has been shown in *Gryllus firmus* (Mole & Zera, 1994; but see Clark et al., 2016) and *Gryllus lineaticeps* (Treidel et al., 2021), but not in *Gryllus rubens* or *Gryllus assimilis* (Mole & Zera, 1993; Zera et al., 1998). Less studied is whether SW crickets exhibit greater reproductive investment because they more efficiently allocate ingested resources to ovary mass, or because they simply ingest more food thereby facilitating increased allocation to reproductive tissue. Understanding resource dynamics in *Gryllus* crickets may inform the evolution of flightlessness, which is fairly common in some insect taxa due to the reproduction‐related costs associated with flight (Guerra, 2011; Roff, 1984; Roff & Fairbairn, 1991). The loss of flight capacity may be favored if flight‐capable crickets exhibit a more precarious resource strategy whereby high metabolic rate and reduced efficiency in allocation of ingested resources to reproduction is not offset by increased food consumption. Thus, I used the variable field cricket (*G. lineaticeps*) to examine the role of flight capacity in (1) circadian patterns of SMR, (2) food consumption, and (3) efficiency by which ingested food is converted to somatic and reproductive tissues. Careful examination of the role of life‐history strategy in circadian patterns of resource use, as well as in resource acquisition and allocation, may inform drivers of life‐history evolution given the increasing uncertainty of food availability in many global regions (Ciais et al., 2005; Currano et al., 2008; Masoero et al., 2020).

## 2 MATERIALS AND METHODS

### 2.1 Study species

The variable field cricket (*G. lineaticeps*) is native to the western United States and is found predominately in California, USA, and Baja, Mexico (Weissman & Gray, 2019). *G. lineaticeps* is wing‐dimorphic like other *Gryllus* crickets (Weissman & Gray, 2019). Both LW and SW wing morphs were used in the study, and they were acquired from a long‐term colony that was subsidized annually by progeny from females collected from a natural population (Sedgwick Reserve). Breeding individuals in the colony were maintained at even sex and morph ratios. Throughout ontogeny, crickets were reared in standard conditions: 28 ± 1°C and 14:10 light:dark cycle with ad libitum access to water (water‐filled shell vials plugged with cotton) and shelter (cardboard egg cartons). Crickets were also supplied with ad libitum access to high‐protein (33% crude protein) commercial dry cat food pellets, because SW and LW *G. lineaticeps* fed a high‐protein diet exhibit the same investment into flight muscle and ovary mass as SW and LW crickets fed a diet of their choice (i.e., when offered several diets ranging in protein‐to‐carbohydrate ratios; Treidel et al., 2021). Newly molted female adults (<1 day after final ecdysis; Day 1) were weighed and individually housed in standard conditions (see above) in small translucent deli cups (473 ml) containing shelter (overturned 30 ml opaque containers with access holes) and preweighed food (pellets of dry cat food).

### 2.2 Experimental design

A factorial design was used to determine the independent and interactive effects of flight capacity and time‐of‐day on SMR, as well as the role of flight capacity in investment into reproductive tissue (i.e., ovary mass), food consumption, and the efficiency by which ingested food was converted to body and ovary mass. Crickets (*n* = 162) were placed into incubators (Model I‐30, Percival Scientific, Inc.) maintaining 28°C and a 14:10 light:dark cycle for 4 days. On Day 5, each cricket and its food were reweighed. Crickets (but not their food) were returned to their individual containers for 24 h before respirometry trials (see below) to limit the confounding effect of feeding state on metabolic rate (Secor, 2009).

On Day 6, each cricket was reweighed before its respirometry trial, which occurred in the morning (within initial 2–3 h of photophase; “AM”) or the afternoon (within final 2 h of photophase; “PM”). To estimate circadian rhythmicity of SMR, trials were performed at these timepoints for two reasons. First, circadian patterns of hormone levels and gene expression vary between the wing morphs when tissue sampling occurs at these two timepoints in at least four other *Gryllus* species in the field and lab, including those that exhibit flight in the scotophase (Zera et al., 2007, 2018; Zhao & Zera, 2004). Second, some field crickets are diurnal and even nocturnal field crickets exhibit some degree of behavioral activity during the photophase—thus, circadian shifts in physiology may be captured during the photophase (Jacot et al., 2008; Levy et al., 2021; Loher, 1979; Rost & Honegger, 1987; Sokolove, 1975; Tomioka & Chiba, 1989; Zera et al., 2007, 2018; Zhao & Zera, 2004). The activity patterns of adult *G. lineaticeps* are less understood. In the field, some female *G. lineaticeps* can exhibit flight activity in the scotophase (Sun et al., 2020), but females in the lab spend an estimated >90% of the scotophase under shelter when housed in isolation (Stahlschmidt et al., in review).

### 2.3 SMR

On Day 6, SMR was determined using flow‐through respirometry (see Supporting Information) as described previously (Padda et al., 2021; Stahlschmidt & Glass, 2020). Carbon dioxide production rate (*V̇*_CO2_) was measured as an indirect estimate of SMR (Clark et al., 2016; Lighton, 2008; Nespolo et al., 2005), because CO_2_ analyzers are typically more sensitive than O_2_ analyzers (Harrison et al., 2012), and *V̇*_CO2_ and *V̇*_O2_ data in this study strongly correlated with one another (*R* = 0.91; *p* < 0.001). Each cricket (*n* = 149) was placed into a small glass metabolic chamber (59 ml) in an incubator (I‐30, Percival Scientific, Inc.) maintaining a constant 28°C. To reduce movement or activity during trials, a strip of opaque tape (width: 2.5 cm) was wrapped around each metabolic chamber to create a darkened (but not light‐free) environment and crickets were acclimated to their chambers for 90 min before measurement.

### 2.4 Resource acquisition and allocation

Food (dry cat food pellets) consumption was determined by subtracting final (Day 5) food mass from initial (Day 1) food mass. To determine the ability of crickets to convert ingested food into body tissue, food conversion efficiency for body mass was estimated (body mass gained [mg]/total food ingested [mg]). On Day 6, crickets were euthanized and stored at −20°C. After storage, crickets were dissected, and their ovaries were removed and dried at 55°C to a constant mass to estimate investment into reproduction (Crnokrak & Roff, 2002; Glass & Stahlschmidt, 2019; Roff & Fairbairn, 1991). Newly molted *G. lineaticeps* exhibit very little investment into reproduction—dry ovary mass is ~0.57% of Day 1 body mass (Stahlschmidt et al., in review). Thus, food conversion efficiency for ovary mass was estimated (estimated dry ovary mass gained between Day 1 and Day 6 [mg]/total food ingested [mg]). During dissections, flight musculature (dorso‐longitudinal muscle [DLM]) was also examined. In other *Gryllus*, LW crickets with histolyzed DLM (LW[h]) are more physiologically similar to SW crickets relative to LWs exhibiting functional DLM (LW[f]; Zera & Larsen, 2001; Zera et al., 1997; reviewed in Zera et al., 2018). Therefore, differences due to flight morphology (i.e., among LW[f], LW[h], and SW crickets) were analyzed for all dependent variables.

### 2.5 Statistical analyses

Data were tested for normality, natural logarithm‐transformed when necessary to achieve normally distributed residuals, and analyzed using SPSS (v.26 IBM Corp.). Two‐tailed significance was determined at *α* = 0.05. To examine the independent and interactive effects of time‐of‐day (morning or afternoon) and flight morphology (LW[f], LW[h], or SW) on metabolic rate, a linear model analysis was performed on body mass‐corrected *V̇*_CO2_ (ml/h/g/Day 6 body mass). Several additional linear models were also performed to examine the effects of flight capacity on food consumption, dry ovary mass, and conversion rates for body mass and dry ovary mass. To account for body size, initial (Day 1) body mass was included as a covariate for the food consumption, final body mass, and ovary mass models.

## 3 RESULTS AND DISCUSSION

### 3.1 Results

**Figure 1:**
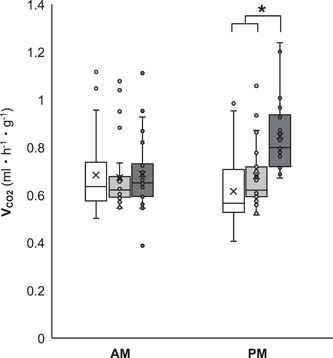
Effects of time‐of‐day (AM or PM) on standard metabolic rate in female *G. lineaticeps* exhibiting variation in flight morphology—short‐winged (SW, white symbols) and long‐winged with histolyzed flight muscle (LW[h], light gray symbols) crickets were flight‐incapable, whereas LW(f) (dark gray symbols) exhibited functional flight muscle (*n* = 149). Asterisk denotes significant difference (*p* < 0.05).

**Figure 2:**
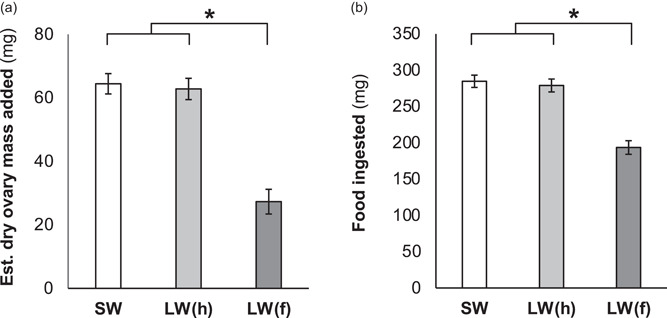
Variation in (a) estimated ovary mass added and (b) food ingested during early adulthood in female *G. lineaticeps* exhibiting variation in flight morphology—short‐winged (SW) and long‐winged with histolyzed flight muscle (LW[h]) crickets were flight‐incapable, whereas LW(f) exhibited functional flight muscle (*n* = 162). Values are displayed as estimated marginal mean ± SEM, because initial (Day 1 of adulthood) body mass was used as a covariate to account for body size. Asterisks denote significant differences (*p* < 0.05).

**Figure 3:**
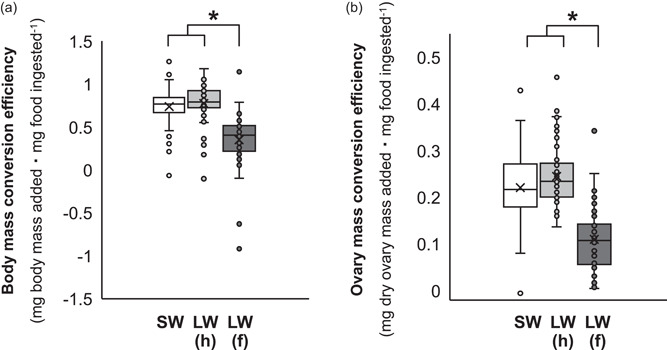
Variation in the efficiency by which ingested food was converted to (a) body mass and (b) dry ovary mass during early adulthood in female *G. lineaticeps* exhibiting variation in flight morphology—short‐winged (SW) and long‐winged with histolyzed flight muscle (LW[h]) crickets were flight‐incapable, whereas LW(f) exhibited functional flight muscle (*n* = 162). Asterisks denote significant differences (*p* < 0.05).

Flight capacity increased energy use due to elevated SMR in the afternoon (i.e., significant effects of flight morphology and a flight morphology × time‐of‐day interaction), but time‐of‐day did not independently influence SMR (Figure 1 and Table S1). Flight capacity reduced reproductive investment and food ingestion (Figure 2 and Tables S2 and S3), as well as the efficiency by which ingested food was converted to body mass and reproductive tissue (Figure 3 and Tables S4 and S5).

## 4 DISCUSSION

An animal must carefully acquire, use, and allocate resources, and this balancing act may be influenced by the animal's circadian clock and life‐history strategy. Here, the wing‐dimorphic *G. lineaticeps* was used to investigate the role of life‐history strategy (investment into flight capacity or reproduction during early adulthood) in resource (food) acquisition and allocation, as well as in circadian patterns of energy use. Flight capacity increased SMR, but this effect of life‐history strategy was due to greater SMR by flight‐capable crickets at the end of the photophase (Figure 1 and Table S1). Flight‐capable crickets did not offset their higher energy use by acquiring or more efficiently allocating food. Relative to flight‐incapable crickets, LW(f) crickets ate less food (Figure 2b and Table S3) and were less efficient at converting ingested food into body or ovary mass (Figure 3 and Tables S4 and S5). In sum, investment into flight capacity for *G. lineaticeps* may represent a risky strategy in some environments, because it reduces fecundity (Figure 2a and Table S2) and the amount of resources available for allocation to other life‐history traits.

Many animals exhibit circadian patterns of energy use, but the degree of circadian rhythmicity in metabolic rate varies significantly across taxa. For example, mammals exhibit fairly consistent circadian patterns in basal metabolic rate (BMR; reviewed in Mortola & Lanthier, 2004). Yet, bird taxa vary in metabolic rhythmicity—passerine birds generally show circadian effects on BMR while many polar birds, sea birds, pigeons, and birds of paradise do not (reviewed in Ellis and Gabrielsen, 2019). Among ectotherms, circadian patterns in metabolic rate are displayed by aquatic invertebrates (Chacón et al., 2003; Rosas et al., 1992; but see Diawol et al., 2016), by fishes (Kim et al., 1997; Ross & McKinney, 1988; Svendsen et al., 2014), and by reptiles (Birchard et al., 2006; Blem & Killeen, 1993; da Cruz et al., 2008; Klein et al., 2006; Roe et al., 2004, 2005). In terrestrial arthropods, circadian rhythmicity in SMR has been exhibited by insects (Banks et al., 1975; Crozier, 1979; Shipp & Otton, 1976; Woodring & Clifford, 1986), spiders (Humphreys, 1977; but see Stoltey & Shillington, 2009), and crustaceans (Reddy & Bhagyalakshmi, 1994; Van Senus, 1985). In this study, flight‐capable (LW[f]), but not flight‐incapable (SW and LW[h]), *G. lineaticeps* exhibited circadian rhythmicity in SMR (Figure 1). Future work is required to determine if this discrepancy is (1) due to the sampling protocol used (e.g., SMR was only examined during the photophase and some field crickets are more active during the scotophase; Sokolove, 1975; Tomioka & Chiba, 1989), or (2) because the evolution of flightlessness may blunt circadian patterns of physiology (Zera et al., 2018; Zhao & Zera, 2004).

Flight capacity led to increased energy use in *G. lineaticeps* (Figure 1) as has been previously demonstrated in *Gryllus* (Nespolo et al., 2008; Sun et al., 2020; Zera & Mole, 1994; Zera et al., 1997; but see Clark et al., 2016). The metabolic cost of flight investment was due to elevated SMR late in the photophase, putatively to facilitate nocturnal flight behavior, which is very metabolically demanding (Roff, 1991). However, metabolic differences due to flight morphology alone (i.e., independent of flight behavior) may be exacerbated in nature where crickets can thermoregulate—in *G. lineaticeps*, LWs exhibit a preferred temperature of 36°C whereas SWs prefer 32.5°C (Sun et al., 2020). Metabolic rate is highly sensitive to temperature and the respiratory quotient for *V̇*_CO2_ in *G. lineaticeps* is 2.4 (Sun et al., 2020). Therefore, differences in thermal preference (Sun et al., 2020) may result in flight‐capable *G. lineaticeps* exhibiting 50% higher SMR than their flight‐incapable counterparts based on SMR data from the current study. Similar to wing morphs, ecotypes (different populations that are genetically distinct) often differ in life‐history strategy and metabolic rate (Andere et al., 2002; Chung et al., 2021; Gangloff et al., 2015; Rouleau et al., 2010). Thus, variation in patterns of energy use may be a fundamental feature of life‐history evolution, although work across taxa paints a fuzzier picture (e.g., metabolic data do not appear to support the pace‐of‐life hypothesis: reviewed in Royauté et al., 2018). As metabolic rate is complex (Glazier, 2015), understanding SMR in the context of life‐history evolution may require incorporating circadian effects. I encourage others to similarly integrate the effects of life‐history strategy (e.g., morph or ecotype) and circadian patterns on SMR.

In *Gryllus*, divergent resource allocation strategies underlie the two life‐history strategies (i.e., the flight‐fecundity tradeoff). Flight‐capable crickets invest in lipogenesis to produce lipid flight fuel and allocate more to somatic stores, whereas flight‐incapable crickets conserve protein and allocate more to ovaries (reviewed in Zera, 2005). These allocation strategies are achieved through differential feeding strategies—flight‐incapable females preferentially feed on a more protein‐biased diet relative to flight‐capable females (Clark et al., 2013; Treidel et al., 2021). Feeding in house (*Acheta*) crickets largely occurs at night (Woodring & Clifford, 1986), but it is not known if flight capacity in *Gryllus* influences circadian patterns of resource acquisition similar to the flight‐dependent rhythmicity of energy use (Figure 1). Although my study used a protein‐biased diet and did not monitor the allocation of specific macronutrients, its results mirror those from a recent study in *G. lineaticeps* that focused on macronutrient acquisition and allocation. Despite burning more calories, flight‐capable *G. lineaticeps* in this study ate 45% less food than flight‐incapable crickets in agreement with Treidel et al., 2021, and they were less efficient at converting ingested food into body or ovary mass (Figures 2b and 3). Therefore, conventional studies using gravimetric data can complement experiments examining the role of macronutrient composition of food in feeding behavior and allocation.

Other *Gryllus* appear to exhibit somewhat different dynamics of resource acquisition and allocation. There is mixed support that LW *G. firmus* eat more than their SW counterparts (Clark et al., 2015; Mole & Zera, 1994) and there is no evidence of flight‐related differences in feeding in *G. rubens* (Mole & Zera, 1993) or *G. assimilis* (Zera et al., 1998). However, flight capacity in four *Gryllus* species, including *G. lineaticeps*, reduces the efficiency by which ingested food is converted into body mass (Figure 3a; Mole & Zera, 1993, 1994; Zera et al., 1998) and flight‐capable *G. lineaticeps* are >40% less efficient at converting ingested food into reproductive tissue (Figure 3b). Thus, flight capacity diverts resources from body stores and reproductive tissue while also increasing energy use, which appears to leave LW(f) crickets in a very precarious energetic state. However, recent work in *G. lineaticeps* indicates that LW(f) crickets eat more food before adulthood relative to flight‐incapable crickets (Treidel et al., 2021). This temporal pattern of feeding may facilitate flight capacity by (1) fueling an increase in body size and features of flight morphology, including wing length and flight muscle mass, before adulthood, because such traits are fixed in size during adulthood, and (2) reducing flight load (body mass) during the dispersal phase of adulthood. Given the mass devoted to reproductive tissue and to filling the gastrointestinal tract, limiting feeding and reproductive investment by LW(f) females significantly reduces overall body mass—flight capacity reduced body mass by nearly 20% in the present study after controlling for body size (femur length). This, in turn, would promote the ability of LW(f) females to disperse away from low‐resource habitats (e.g., patches characterized by suboptimal temperatures, or limited food or mates).

The costs and benefits of flight investment vary across taxa (reviewed in Dingle, 2014). In *Gryllus*, the relative advantages of flight capacity are environment‐dependent (e.g., Glass & Stahlschmidt, 2019; Padda et al., 2021) and stable environments are predicted to favor the evolution of flightlessness in insects (Roff & Fairbairn, 1991; Roff, 1984). However, periods of food insecurity are expected to change in frequency and duration with global climate change (Ciais et al., 2005; Currano et al., 2008), and food may play an important role in the evolution of flightlessness in *Gryllus*. For example, a stable, constant supply of food during development reduces flight capacity in *G. firmus* (Glass & Stahlschmidt, 2019), and a greater intake of food and protein, in particular, is associated with reduced flight capacity in *G. lineaticeps* (Treidel et al., 2021). Flight capacity in *G. firmus* and *G. lineaticeps* also affects protein and carbohydrate regulation strategies (Clark et al., 2013, 2015, 2016; Treidel et al., 2021), and the effect of climate change on the quality or macronutrient composition of food may alter the relative advantages of different life history strategies (Lenhart, 2017). Ongoing climate change may also affect the evolution flightlessness in *Gryllus* via shifts in temperature. Warmer temperatures facilitate flying behavior (Sun et al., 2020) and the expression of the LW morph (Roff & Fairbairn, 2007), and flight capacity is also favored by a stable developmental temperature (Glass & Stahlschmidt, 2019). Therefore, wing‐dimorphic crickets may prove to be a valuable system to examine the roles of food, temperature, and circadian patterns in life‐history evolution in a changing world.

## CONFLICT OF INTEREST

The author declares no conflict of interest.

## Supporting information

Supplementary information.

## Data Availability

All data are available upon request.
