# Supplementary material for: Flight capacity drives circadian patterns of metabolic rate and alters resource dynamics
Source: J Exp Zool A Ecol Integr Physiol. 2022 Apr 19;337(6):666–74. doi: 10.1002/jez.2598 (PMC9324922; doi:10.1002/jez.2598)
Supplement: Supplementary file 1 — Supplementary information. [file JEZ-337-666-s001.docx]

**Supplemental Information**

*Flow-through respirometry*

Seven metabolic chambers at a time were connected to a multiplexer (MUX, Sable Systems, Las Vegas, NV, USA), and an eighth port on the multiplexer allowed for baseline measurements of influent air throughout the 86 min. sampling period. Influent air was generated by a diaphragm pump (PP2, Sable Systems, Las Vegas, NV, USA), and flow rates were regulated by needle valves and monitored by mass flow meters (FB8, Sable Systems, Las Vegas, NV, USA). The multiplexer passed one air stream sequentially through each chamber for 10 min. for respiratory measurements (70 ml ∙ min.^−1^ per chamber), while the other air stream provided continuous air to crickets during non‐measurement periods (~40 ml ∙ min.^−1^ per chamber). Influent air was stripped of H_2_O and CO_2_ by flowing it through columns of CaSO_4_ and soda lime, respectively. Sample effluent air was first passed through a water vapor meter (FMS, Sable Systems, Las Vegas, NV, USA), after which water was removed via CaSO_4_. Air then passed through a CO_2_ analyzer (FMS, Sable Systems, Las Vegas, NV, USA). All hardware (e.g., FMS and FB8) interfaced with a software (ExpeData, Sable Systems, Las Vegas, NV, USA) that recorded data each second to a computer. Each cricket's steady-state *V̇*_CO2_ was determined from the average of the most level 5 min. sequence (i.e., 300 samples) of data (Lighton, 2008).

*Reference*

Lighton, J. R. (2008). Measuring Metabolic Rates: A Manual for Scientists. Oxford: Oxford University Press.

*Supplemental tables*

Table S1: Effects of time-of-day (AM or PM) on mass-specific standard metabolic rate in female *G. lineaticeps* exhibiting variation in flight morphology—short-winged crickets, long-winged crickets with histolyzed flight muscle, and long-winged crickets with functional flight muscle (n=149).

|  | df | F | P |
| --- | --- | --- | --- |
| Intercept | 1,143 | 5804.5 | <0.001 |
| Time-of-day | 1,143 | 1.7 | 0.19 |
| Flight morphology | 2,143 | 8.7 | <0.001 |
| Time*Morph | 2,143 | 7.9 | 0.001 |

Table S2: Effects of flight morphology—short-winged crickets, long-winged crickets with histolyzed flight muscle, and long-winged crickets with functional flight muscle—on estimated ovary mass added during early adulthood in female *G. lineaticeps* (n=162). Initial (<1 d after final ecdysis) body mass was included as a covariate to account for body size.

|  | df | F | P |
| --- | --- | --- | --- |
| Intercept | 1,121 | 0.3 | 0.6 |
| Flight morphology | 2,121 | 32.7 | <0.001 |
| Initial body mass | 1,121 | 28.2 | <0.001 |

Table S3: Effects of flight morphology—short-winged crickets, long-winged crickets with histolyzed flight muscle, and long-winged crickets with functional flight muscle—on total amount of food (dry cat food pellets) consumed during early adulthood in female *G. lineaticeps* (n=162). Initial (<1 d after final ecdysis) body mass was included as a covariate to account for body size.

|  | df | F | P |
| --- | --- | --- | --- |
| Intercept | 1,157 | 31.6 | <0.001 |
| Flight morphology | 2,157 | 31.7 | <0.001 |
| Initial body mass | 1,157 | 13.7 | <0.001 |

Table S4: Effects of flight morphology—short-winged crickets, long-winged crickets with histolyzed flight muscle, and long-winged crickets with functional flight muscle—on the conversion of ingested food (dry cat food pellets) into body tissue during early adulthood in female *G. lineaticeps* (n=162).

|  | df | F | P |
| --- | --- | --- | --- |
| Intercept | 1,158 | 977.5 | <0.001 |
| Flight morphology | 2,158 | 43.1 | <0.001 |

Table S5: Effects of flight morphology—short-winged crickets, long-winged crickets with histolyzed flight muscle, and long-winged crickets with functional flight muscle—on the conversion of ingested food (dry cat food pellets) into reproductive tissue during early adulthood in female *G. lineaticeps* (n=162).

|  | df | F | P |
| --- | --- | --- | --- |
| Intercept | 1,122 | 847 | <0.001 |
| Flight morphology | 2,122 | 20 | <0.001 |
